# The effectiveness of sedentary behaviour interventions on sitting time and screen time in children and adults: an umbrella review of systematic reviews

**DOI:** 10.1186/s12966-020-01009-3

**Published:** 2020-09-21

**Authors:** Phuong Nguyen, Long Khanh-Dao Le, Dieu Nguyen, Lan Gao, David W. Dunstan, Marj Moodie

**Affiliations:** 1 Deakin Health Economics, Institute for Health Transformation, Deakin University, Geelong, Victoria Australia; 2 Global Obesity Centre, Institute for Health Transformation, Deakin University, Geelong, Victoria Australia; 3 School of Biomedical Sciences and Pharmacy, The University of Newcastle, Callaghan, NSW Australia; 4 Baker Heart and Diabetes Institute, Melbourne, Australia; 5 Mary MacKillop Institute for Health Research, Australian Catholic University, Melbourne, Victoria Australia

**Keywords:** Systematic review, Sedentary behaviour, Effectiveness, Sitting time, Screen time, Sedentary time

## Abstract

**Background:**

There is increasing concern about the time people spend in sedentary behaviour, including screen time, leisure and occupational sitting. The number of both primary research studies (published trials) and reviews has been growing rapidly in this research area. A summary of the highest level of evidence that provides a broader quantitative synthesis of diverse types of interventions is needed. This research is to articulate the evidence of efficacy of sedentary behaviour interventions to inform interventions to reduce sitting time. The umbrella review, therefore, synthesised systematic reviews that conducted meta-analyses of interventions aiming at reducing sedentary behaviour outcomes across all age group and settings.

**Method:**

A systematic search was conducted on six databases (MEDLINE Complete, PsycINFO, CINAHL, Global Health via EBSCOhost platform, EMBASE, and Cochrane Central Register of Systematic Reviews). Included articles were systematic reviews with meta-analysis of interventions aiming at reducing sedentary behaviour (screen time, sitting time or sedentary time) in the general population across all age group.

**Results:**

Seventeen reviews met the inclusion criteria (7 in children and adolescent, 10 in adults). All reviews of sedentary behaviour interventions in children and adolescents investigated intervention effectiveness in reducing screen time. Six out of 11 meta-analyses (reported in 7 reviews) showed small but significant changes in viewing time. All reviews of sedentary behaviour interventions in office workplaces indicated substantial reduction in occupational sitting time (range: 39.6 to 100 min per 8-h workday). Sub-group analyses reported a trend favouring environmental change components such as sit-stand desks, active permissive workstations etc. Meta-analyses indicated that sedentary behaviour interventions were superior to physical activity alone interventions or combined physical activity and sedentary behaviour interventions in reducing sitting time.

**Conclusion:**

The current systematic reviews and meta-analyses supported sedentary behaviour interventions for reducing occupational sitting time in particular, with small changes seen in screen time in children and adolescents. Future research should explore approaches to maintaining behaviour change beyond the intervention period and investigate the potential of sedentary behaviour reduction interventions in older age groups in non-occupational settings.

## Background

Sedentary behaviour (SB), defined as any posture (sitting, reclining or lying) characterised by an energy expenditure ≤1.5 metabolic equivalents of task (MET) while waking [1], is observed in all domains including behaviours at work or school, at home, during transport, and in leisure-time. Examples are watching television, playing board games, driving or sitting whilst travelling, sitting or lying down, whilst reading, or sitting at work (desk-based computer) [2]. Of particular importance is the evidence of a steady increase in sedentary occupations research, which has emerged over the last few decades [3]. Specifically, evidence from developed countries (using accelerometers) indicates that sedentary time ranges between 55% and 70% of adult waking hours [4], with an average sedentary time of ≥9 h per day [5]. Children aged 2–4-years spend on average almost 1.5 h per day on sedentary activities such as watching TV/DVDs or playing electronic games, while 5–17 years spend over 2.25 h per day [6]. Both age groups had higher levels of SB than recommended by Australian guidelines [7]. The recommendation to limit sedentary recreational screen time to no more than 2 h per day in children and adolescents is consistent across guidelines [8, 9]. However, in adults, there are no recommendations for maximum daily SB time or the frequency of sitting breaks [9, 10].

There is strong evidence that high amounts of SB increase the risk for all-cause [10–12] and cardiovascular disease (CVD) mortality [10, 12] and incident CVD [10] and type 2 diabetes (T2D) in adults [10, 12]. Furthermore, moderate evidence indicates that SB is associated with incident endometrial, colon and lung cancer [10, 13]. The hazardous effects of SB are more pronounced in physically inactive people [10, 14]. In children, longer duration and higher frequencies of screen time negatively impact on body composition, cardiometabolic risk, behaviour, fitness and self-esteem [15].

The problem of too much sitting has been increasingly recognised through public health guidelines that now incorporate explicitly, yet rather broad, messages around SB [8–10]. Given the high exposure to SB and the negative impacts on population health, research in this area has gained prominence over the last 10 years [16], leading to the conduct of intervention studies targeting reductions in SB in different contexts and diverse population groups. As a consequence, a number of systematic reviews have been published in recent years to quantify the impact of SB interventions on measures of sitting time, with most predominantly focusing on workplace interventions and some community-based interventions.

Decision-makers and policymakers are increasingly favouring approaches of summarising the ‘totality’ of the evidence on effectiveness to inform practice and guidelines. In relation to SB, a review of systematic reviews, as a result, would be valuable in providing a high level of synthesised evidence to inform decision-makers about intervention effectiveness. To our knowledge, only one review of systematic reviews has been conducted to date to evaluate the effectiveness behaviour change interventions designed to reduce SB due to TV watching and/or media use in children and adolescents [17]. That umbrella review suggested future research should evaluate interventions targeting other types of SB and other population age groups (e.g. adults), as well as the effectiveness of different behaviour change techniques across different settings [17]. This umbrella review sets out to investigate the efficacy of interventions in reducing SB in healthy populations across all age groups. To contrast and compare the effectiveness of intervention components or strategies (quantified by effect size), a review of systematic reviews that included meta-analysis was conducted.

The aim of this umbrella review (a review of reviews that conducted meta-analyses) was to (a) assess the effectiveness of SB interventions on measures of overall sitting time, occupational sitting time and screen time; (b) examine the effectiveness of specific intervention components; and (c) identify whether there is a research gap in terms of the potential types of interventions targeting SB.

## Method

This review adheres to the guidelines in the PRISMA statement 2009 [18] in combination with Joanna Briggs Institute (JBI) guidelines for umbrella reviews [19]. The protocol was registered with PROSPERO: International Prospective Register of Systematic Review Protocols (registration number CRD42020150458).

### Search strategies and databases

A literature search for potentially eligible publications was conducted by the first author (PN) in collaboration with an experienced librarian and the review team. The data search was conducted on 29th August 2019 using six bibliographic databases: MEDLINE Complete, PsycINFO, CINAHL, Global Health via EBSCOhost platform, EMBASE and Cochrane Central Register of Systematic Reviews. Search strategies were developed based on three concepts: SB, intervention and study type. The following search terms were modified to reflect subject headings control (or medical subject heading - MeSH terms) that fit individual database searches (details in Additional file 1).

(“sedentary behavio*” OR “sedentary lifestyle*” OR “sedentary time” OR “sedentary activit*” OR “sedentary leisure” OR sitting OR “seated posture” OR “screen time” OR “computer time” OR ((watch* OR view*) N2 (tv OR television)) OR inactive*) AND (effect* OR efficac* OR evaluat* OR intervention* OR program* OR compar*) AND (“systematic review” OR meta-analys* OR meta-analytic* OR “quantitative analys*”)

### Inclusion and exclusion criteria

The umbrella review sought to identify all reviews of studies that examined the effects of interventions that aimed to reduce SB and reported behaviour outcomes, e.g. sedentary time, sitting time and sedentary screen time across the age spectrum. Reviews of weight loss or physical activity (PA) interventions that also aimed to reduce sedentary time by incorporating a SB reduction component and reported SB outcomes were included. For inclusion, studies were required to be (a) a systematic review that included a meta-analysis; (b) a review that included randomised controlled trials (RCTs), with or without other types of studies. Whilst there was no restriction placed on the participants’ gender and age groups, studies were limited to the general population, i.e. reviews that included trials targeting specific health conditions such as CVD, T2D etc. were thus excluded. However, reviews of SB interventions for the general population that did not exclude people with specific conditions were included. The search was limited to human studies reported in English with no date restriction.

### Identification of relevant studies and data extraction

All citations were imported into Endnote and duplicates were removed using both the Endnote function and manually. Reviews were selected by (a) screening of title and abstract; (b) full-text screening independently by three review authors (PN, LL and DN) using Rayyan web-app for systematic reviews [20]. Discrepancies resulting from the screening were resolved based on consensus amongst the review team.

Overlap of primary research studies in each of the included reviews was identified by comparing the list of included primary studies in each review.

For reviews that met the inclusion criteria, data extraction and quality assessment were conducted independently by two team members (PN and LL, PN and DN). Extracted information included the characteristics of the reviews (databases, number of included trials, search date and range of publication year); sample (total size, age); characteristics of primary studies (type of study, setting, country); intervention description (components, control, SB measurement) and intervention effectiveness results (as reduction in time spent in SB, e.g. sitting time, screen time, total SB time). The primary outcome of interest of this umbrella review was the intervention effect size (post-intervention change-from-baseline difference between intervention and control group) reported in the quantitative analyses.

### Quality assessment

Bias and quality of the included systematic reviews and meta-analyses were assessed using A Measurement Tool to Assess Systematic Reviews (AMSTAR) [21]. This tool consists of 16 items that assess both methodological quality and reporting quality. The overall rating of high, moderate, low and critically low, is based on weaknesses or flaws in ten critical domains [21, 22].

## Results

**Fig. 1:**
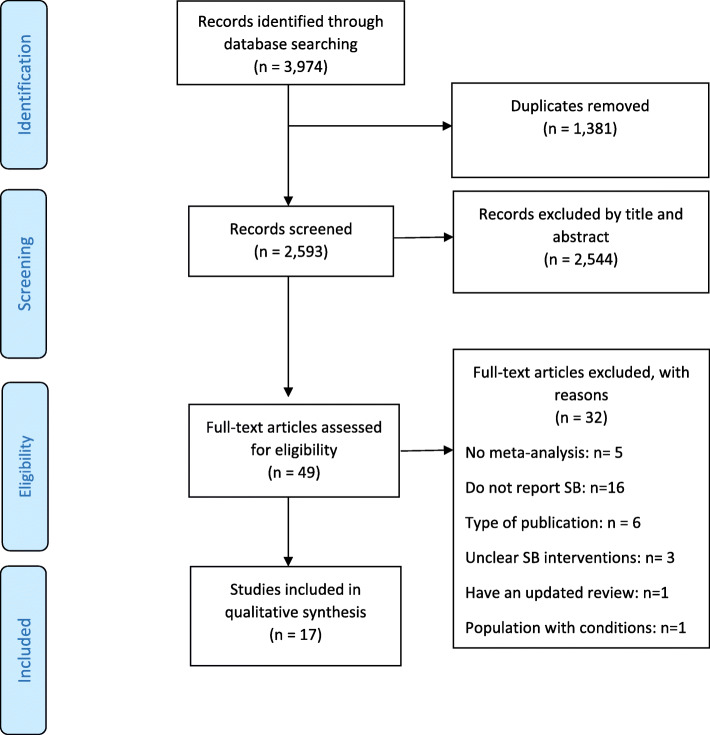
PRISMA flowchart

The literature search yielded 3974 titles; of these, 17 systematic reviews with meta-analysis [23–39] were eligible for review (Fig. 1).

Of the 17 included reviews, six reviews examined intervention effectiveness in children and adolescents across various settings [23, 26, 27, 31, 32, 35], mainly schools and home. Three reviews investigated SB interventions targeting adults in office workplaces [24, 28, 29], whilst the other six reviews explored interventions targeting adults across all settings [30, 34, 36–39]. Two reviews evaluated SB interventions across all age groups [25, 33]; however, only one review conducted sub-groups analysis for each age group [33]. Characteristics of the included reviews are presented in Additional file 2.

Behaviour outcomes reported in the included reviews were screen time (measured using self-report), sitting time (measured through self-report or device-measured detecting sit-stand posture) and sedentary time (measured by a device such as an accelerometer that detects intensity level). Screen time was the dominant outcome reported in children and adolescent, and it was reported in all seven studies in this age group, with only one study also investigate total sitting time [26] (Table 1).

**Table 1:** Summary of evidence from quantitative research syntheses

SB Interventions	Author/year	Effectiveness^#^ Mean (95% CI)		
		Screen time	Occupational sitting and/or other sitting time	Overall sitting time with/ without screen time
Children	Biddle 2011 [23]	− 0.19 (− 0.30; − 0.08)^%^		
	Downing 2018 [26]	−17.12 min/d (− 28.82; − 5.42)		−18.91 min/d (− 33.31; − 4.51)
	Grieken 2012 [31]	−17.95 min/d (− 26.61; − 9.28)		
	Kamath 2008 [27]	−0.31 (− 0.38, − 0.24) ^%^		
	Maniccia 2011 [35]	**−0.10 (− 0.48; 0.27)** ^**%;a**^ − 0.13 (− 0.24; − 0.01) ^%;b^		
	Wahi 2018 [32]	**−0.90 h/wk (− 3.47; 1.66)**		
	Wu 2016 [33]	**−2.99 h/wk (− 7.51, 1.52)**		
Adolescents	Grieken 2012 [31]	No sub-group analysis		
	Kamath 2008 [27]	**0.00 (− 0.25, 0.25)** ^**%**^		
	Maniccia 2011 [35]	−0.18 (− 0.30; − 0.05) ^%^		
	Wu 2016 [33]	**−3.04 h/wk (− 7.62, 1.54)**		
Adults	Compernolle 2019 [34]		−0.56 (− 0.90; − 0,07) ^%^	−0.32 (− 0.50; − 0,14) ^%^
	Chu 2016 [24]		− 39.6 min/8-h (− 51.7; − 27.5)	
	Direito 2016* [25]			−0.26 (− 0.53; − 0.00) ^%^
	Neuhaus 2014 [28]		−77 min/ 8-h (− 120; − 35)	
	Martin 2015 [36]			−22.34 min/d (− 35.81; − 8.88)
	Peachey 2018 [37]		−29.96 min/d (− 44.05; − 15.87)	−30.37 min/d (− 40.86; − 19.89)
	Prince 2014 [38]			−1.28 (− 1.68; − 0.87) ^%^
	Shrestha 2018 [29]		−100 min/ 8-h (− 116; − 84)	
	Shrestha 2019 [39]	−61.08 min/d (− 79.40 to − 42.76)	−30.18 min/d (− 58.47; − 1.88)	
	Stephenson 2017 [30]			−41.28 min/d (− 60.99; − 21.58)
	Wu 2016 [33]	−14.98 h/wk. (− 16.22, − 13.75)		

Note: a: effect size in children under 5 years; b: effect size in children 5 to 11 years, *h /wk* hour per week, *min/d* minutes per day, *SB* sedentary behaviour, *SMD* standardized mean difference, *95% CI* 95% confident interval

Bold numbers indicate non-significant effect sizes. Negative numbers indicate reduction

#Meta-analyses reported post-intervention change-from-baseline difference between intervention and control group)

*Meta-analysis did not report effect size for adults and children separately

%Standardized mean difference, where effects were measured in different scale

The majority of reviews of SB intervention in adults had a primary focus on reductions in total sitting time (across all domains), with many focusing on reductions to occupational sitting time. Five reviews pooled screen time and sitting time in the primary analysis to report total time spent in sedentary activities [25, 30, 34, 36, 38]. When sitting time was used as the primary outcome, but not limited to occupational sitting, then the term “sedentary time” was interchanged. There was a significant overlap of primary studies included by Stephenson et al. 2017 [30], examining the effectiveness of technologies to reduce sedentary time, i.e. multi-context sitting time with/ without screen time, and by Chu et al. 2016 [24], investigating the effectiveness of SB interventions in reducing occupational sitting time. Summary of research evidence on SB intervention effectiveness is presented in Table 1.

**Fig. 2:**
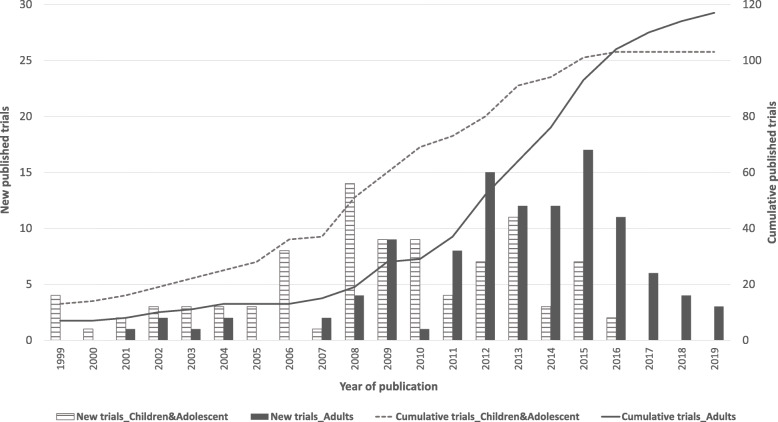
Number of new and cumulative sedentary behaviour intervention trials over the two decades, 1999–2019

Overlapping was also observed in included reviews. Six out of 17 primary studies included by Stephenson et al. 2017 [30] were office workplace interventions. Seven out of 17 studies in Biddle et al. 2011 [23] were also included in Grieken et al. 2012 [31]. However, none of the reviews had an overlapping of more than 40% of its included trials. The 17 reviews included in this umbrella review comprised a total of 219 trials, of which 102 trials targeted children and adolescents, and 117 targeted adults. Figure 2 presents the cumulative total number of SB trials published in the period 1999–2019. Given the insufficient level of detail reported in the included reviews, the number of primary interventions could not be analysed by settings.

### Sedentary behaviour interventions

#### Intervention delivery and components

The majority of the primary interventions were conducted in high-income countries including the United States, the United Kingdom, Australia and Europe. The length of interventions varied from a few days to 4 years, with the longer-term trials tending to be interventions targeting children and adolescents in the school setting. The included reviews did not provide sufficient data to quantitatively summarise intervention setting, except for workplace interventions (3 independent reviews [24, 28, 29] with a total of 42 trials adopting offices as the primary setting).

Intervention components were categorised into three main groups, i.e. motivational/volitional component, physical environmental changes and policy changes [24, 29]. Multi-component interventions were defined differently across reviews. Shrestha et al. [29] defined multi-component intervention as those including all three component categories, whilst Chu et al. [24] defined multi-component as a combination of motivational/volitional strategies and environmental changes. Details of common strategies under each component are presented in Table 2. Motivational/volitional strategies were available across all type of settings, while the other components were limited to specific settings, i.e. policy changes were employed mostly in the office setting, whilst environmental changes were observed in both office and classroom/ school as well as home settings.

**Table 2:** Intervention settings and components of SB interventions

Component	Settings				
	***School***	***Office***	***Home***	***Community***	***Primary care***
***Motivation/volition***	Educational sessions on healthy lifestyle; Mass media or other health promotion materials, e.g. posters, newsletters etc. (7 reviews) [23, 26, 27, 31–33, 35]	Goal setting in sitting time; Provide educational materials and tips via email or message; Strategies for self-monitoring and reinforcement for behaviour change; Motivational interview/ counselling sessions; (8 reviews) [24, 29, 33, 34, 36–39]	Goal setting in screen time; Dairy and materials to promote healthy lifestyle. (10 reviews) [26, 27, 31–36, 38, 39]	Group counselling; Mass media or other health promotion, e.g. posters, newsletters etc. (3 reviews) [23, 26, 36]	Counselling sessions, individualized lifestyle plan; Monitoring handbook (3 reviews) [23, 26, 36]
***Environmental change***	Sit-stand or standing desk (1 review) [26]	Sit-stand workstation, portable elliptical/pedal machine; stationary cycle ergometer and treadmill desk; reminder application installed in computer and tracking devices etc. (8 reviews) [24, 28–30, 34, 36–38]	Remove TV out of bed-rooms; screen time monitoring devices; sitting time monitoring/ tracking devices (7 reviews) [27, 30, 33–35, 38, 39]	Not available	Not available
***Policy change***	Curriculum change (3 reviews) [26, 32, 35]	Walking or standing meetings; organizational schedule for sitting breaks and stand up. (1 review) [29]	Not available	Not available	Not available

##### Intervention delivery and components in children and adolescent

Interventions promoting healthy lifestyles in children overwhelmingly employed motivational/volitional strategies; they also targeted other risk behaviours such as physical activity and diet [17, 27] and were delivered in multiple-settings, i.e. schools in combination with home and/or community. The included reviews of SB interventions in children and adolescents highlighted the involvement of parents, educators and the importance of trainings for parents [26, 27, 31]. Environmental changes component such as sit-stand desks in class were discussed only in one review (see Table 2).

##### Intervention delivery and components in adults

Reviews of occupational sitting (3 out of 11 reviews) were workplace interventions that had targeting workers in office-based occupations [24, 28, 29]. A 2016 review reported that motivational/volitional strategies were the most common components with 15 primary trials employing these strategies, compared to only five trials encompassing physical environment changes [24]. In contrast, a 2014 review [28] reported 20 trials in office settings as employing workplace environmental changes, i.e. sit-stand-desk or adjustable standing desk, active work stations, etc. The most recent Cochrane review of workplace interventions in 2018 [29] identified the most popular strategies as physical changes in the workplace (*n* = 10) followed by motivational/volitional strategies (*n* = 2).

Eight reviews (out of 11 reviews in adults) investigated SB intervention effectiveness in adults across all settings, including both workplace and non-occupational settings (i.e. home, community, primary care, clinics) [25, 30, 33, 34, 36–39]. Of these, two reviews [25, 30] investigated the effectiveness of technologies to reduce SB such as computer prompts, reminder emails or messages, wearable tracking devices, mobile phone etc. were considered as motivational/volitional components.

#### Intervention outcomes

All included reviews highlighted that outcome measurement in the trials was often based on self-reported sitting time and sedentary screen time. Trials that used objective measurement predominately investigated interventions within office settings. The difference in intervention effectiveness between objective measurement and self-report remains inconclusive. Greater effect sizes following objective measurement were reported in one review of occupational sitting [24] and general sedentary time [34]. However, another had a more substantial effect based on self-report [30, 37], whilst another [25] reported a non-significant difference between the two types of measurements.

Only one review attempted to analyse the intervention effects on breaks and the number of prolonged sitting bouts but reported non-significant effects in those two outcomes [34, 36].

#### Intervention effectiveness

##### Intervention effectiveness in children and adolescent

Of the seven reviews investigating SB in children, all except one reported significant, small to moderate effect sizes on reduction in screen time [23, 26, 27, 31–33]. The other study reported a non-significant reduction in screen time mean difference (MD) -0.90 h per week (95% CI − 3.47 to 1.66) [32]. A meta-analysis of sedentary time and screen time together reported the overall MD of − 17.12 min/day (95% CI − 28.82 to − 5.42) and MD of sedentary time between groups as − 18.91 (95% CI − 33.31 to − 4.51) [26]. The quantitative analysis reported in Biddle et al. [23] indicated larger effect sizes following multi-risk behaviour interventions in children. Motivation/volition components tended to be more effective in children [27], while there was no significant difference between single- and multi-component interventions [31]. Sub-group analyses sought to identify the association between length of intervention and effectiveness, but the results remained inconclusive with one review supporting interventions longer than 12 months (long term interventions) [23], while another review reported larger effect sizes following medium-term interventions (4–6 months) [36]. Another two sub-group analyses revealed larger effect sizes following short-term (3 months or less) interventions [23, 27].

Comparing intervention effectiveness by age group, Kamath et al. [27] reported a more substantial and significant effect size in children (standardized mean difference (SMD) -0.31; 95%CI − 0.39 to − 0.24) and a non-significant effect in adolescents (SMD 0.00; 95%CI − 0.25 to − 0.20). In contrast, a subgroup analysis by Maniccia et al. [35] reported a stronger effect size in the adolescent group (g = − 0.176 (95% CI − 0.386 to − 0.049) versus g = − 0.125 (95%CI − 0.241 to − 0.008) in children. Table 1 provides a summary of intervention effect sizes across all age group.

##### Intervention effectiveness in adults

One systematic review [38] with meta-analysis reported the effects of reducing SB by three different types of interventions: PA interventions that aimed to reduce SB; SB combined with PA intervention, and solely SB focused-interventions. The study reported moderate-quality evidence (assessed using Cochrane GRADE framework - Grading of Recommendations, Assessment, Development and Evaluations) that large and clinically meaningful reductions in sedentary time resulted from interventions with a focus on reducing SBs (SMD = − 1.28 [95% CI: − 1.68 to − 0.87]). Another meta-analysis [36] supported this evidence with multi-risk behaviour interventions reducing SB by 24 min/day (95% CI − 41 to − 8 min/day, moderate quality). Meanwhile, interventions focusing on SB resulted in a mean reduction of 42 min/day (95% CI − 79 to − 5 min/day, low quality using Grade). There was no evidence of an effect amongst PA and combined PA/SB interventions on reducing sedentary time [36].

Reductions in sitting time were similar between interventions in workplaces (− 29.96 min/day; 95% CI − 44.05 to − 15.87) and other settings, which included community, domestic and recreational environments (− 30.47 min/day; 95% CI − 44.68 to − 16.26) [37]. However, looking at the level of evidence and effect size, stronger and consistent evidence is more likely to support SB intervention effectiveness in the office setting (Table 1).

For interventions specifically targeting adults in workplace settings, one review reported a more substantial effect size for multi-component interventions (mean difference MD − 88.8 min/8 h workday 95% CI − 132.7 to − 44.9 [24], compared to environmental changes intervention (MD − 72.8 min/8-h workday; 95% CI − 104.9 to − 40.6) and motivation/volition components (MD − 15.5 min/8-h workday, 95% CI − 22.9 to − 8.2) [24]. However, two other reviews concluded that environmental changes components were more effective than the other components. The meta-analysis in the Cochrane review reported that physical workplace changes resulted in a change of − 100 min/8-h working day (95% CI − 116 to − 84), while the effect size of workplace policy changes and counselling components were non-significant [29]. Another review that quantitatively analysed intervention effects on sitting time across all settings reported similar results; environmental interventions had the largest reduction in daily sitting time (− 40.59 min/day; 95% CI − 61.65 to − 19.53), followed by multi-component (− 35.53 min/day; 95% CI − 57.27 to − 13.79) and motivation/volition component (− 23.87 min/day; 95% CI − 37.24 to − 10.49) [37]. Another review reported a reduction of − 77 min/8 h workday following active-permissive workstation component [28].

No evidence was available on the effectiveness of interventions for reducing non-occupational sedentary time in older adults (age > 70 years) [39]. One review identified five interventions targeting older adults [34]; of those, only three trials involved a healthy population, whilst the other two targeted breast cancer survivors and people with T2D. No subgroup analysis was conducted [34].

A review of interventions using technology to reduce time spent in SB in various settings reported an overall reduction in sitting time of − 41.28 min/day (95% CI − 60.99 to 21.58) [30], which is a slightly smaller effect size compared to interventions in the office setting. Another review investigating the effectiveness of technologies in reducing SB in all age group 8–71 years, reported a small effect of − 0.26 h/day (95% CI − 0.53 to 0.00) [25].

##### Methodological issues in trials evaluating the effectiveness of SB interventions

The included reviews highlighted a range of methodological issues of trials for SB interventions. Evidence highlighted that non-random allocation and concealment were generally inevitable for trials evaluating SB interventions in workplaces, communities and schools [26, 29, 30, 32]. Non-continuous measures (percentage) of screen time or sitting time reported in the primary studies raised an issue in the quantitative analyses, making it challenging to compare intervention effectiveness [26]. Issues with self-reported SB were highlighted, such as an unclear description of the self-report methods and measures captured and the lack of standardised methods in measuring screen time [31, 33]. Reviews suggested that while objective measurement is preferred for accuracy and validity, self-report measurements are also beneficial in analysis of SB domains, i.e. behaviours undertaken during sitting [28, 39]. The reviews also highlighted the lack of long-term, large scale and low risk of bias trials [26, 28].

### Quality of included systematic reviews

The quality of the majority of the included reviews was rated as moderate using AMSTAR. Three reviews [29, 38, 39] were rated as high quality, eight as moderate quality [24, 26, 28, 30, 32–34, 37] and six as low quality [23, 25, 27, 31, 35, 36]. Whilst one review confined its literature search to three databases [33], the majority of reviews included five databases [23, 24, 27, 30, 31, 34, 37] or more [25, 26, 28, 29, 32, 35, 36, 38, 39].

For systematic reviews with meta-analysis, it is imperative to describe the controls, but only 11 of the 17 reviews provided information regarding comparator groups [25, 29–31, 33–39]. Different comparators were employed across trials, i.e. do-nothing control or counselling or other types of interventions, however, only seven of the meta-analyses conducted a sub-group analysis for different controls [29, 33–35, 37, 39]. Six reviews had a mixed population, i.e. included both healthy people and people with co-morbidities [34–39], but only one review conducted subgroup analysis for people with conditions [35]. Additionally, some reviews did not provide sufficient data regarding settings in individual primary studies included [25, 34, 38]. It is noted that some meta-analyses [24, 29] reported reduction in occupation sitting per 8-h working day but the analysis also included trials having office as primary setting but reported overall sitting time, i.e. total sitting time per day or per week outside of office hour.

## Discussion

This umbrella review provides a state-of-art level of evidence syntheses of the findings from 17 systematic reviews that conducted meta-analyses of interventions designed to reduce SB across different age groups. This area of research continues to be highly productive, and new trials are emerging at a rapid rate [40]. Moreover, this umbrella review is the first review of reviews making an effort to examine the effectiveness of SB interventions on measures of behaviour change (both screen time and sitting time), and to cover a broader span of interventions. This umbrella review also summarises quantitative evidence regarding the effectiveness of different intervention components and different settings.

With many primary studies published, all the included reviews often addressed different outcomes, or different age groups and/or settings despite some overlap in the primary studies investigated. Since the overlap observed was small, their impact on the results of the quantitative analyses was not explored. Given that data extraction was conducted at the review level, there were some uncertainties around whether the included primary studies were SB intervention per se or PA focused interventions that aimed to reduce SB. This umbrella review, therefore, accepted the reviews’ classification of trials. Lastly, SB interventions targeting the older age group were not captured in this review. A potential reason may be the lack of sufficient data or primary studies to conduct a meta-analysis. One review, included five studies in older people, with only one being an RCT and the rest pilot studies [41]. Another reason is that interventions targeting this age group often focus on participants’ conditions, i.e. stroke survivors, CVDs, cancer, which is outside of the scope of this umbrella review [41].

This evidence synthesis indicated that SB interventions were superior to PA interventions and PA + SB combined in reducing sedentary time. This result is supported by the fact that SB is different from insufficient physical activity, where the latter refers to not meeting the recommended level of PA as per guidelines [1, 42]. Moreover, this SB superiority is probably strongly related to the distinct differences in behavioural barriers/ facilitators of SB and PA. Targeting specific SB behavioural elements is likely to have unique effects. However, this in turn raises the question of how PA components can be incorporated effectively into an SB intervention. The relative benefits of a greater reduction in sitting time being replaced with standing or light-intensity PA compared with a smaller reduction in sitting time being replaced with moderate-vigorous PA warrants further research. Different strategies can achieve comparable benefits, and replacing SB with light-intensity PA might be of value for those individuals who find moderate-vigorous PA challenging [14, 43].

Secondly, our findings support employing environmental changes components to facilitate the reduction in SB. This component was shown to yield the most significant effect size; it was also the most common component for SB interventions, especially in workplace settings [29, 37]. However, analyses indicated that environmental changes such as sit-stand desks in office setting offered greater effects in the short and medium-term.

Interestingly, the effects of motivational/volitional strategies tended to increase in accordance with the length of intervention, i.e. non-significant effect in the short term but a significant reduction at medium-term follow-up [29]. Furthermore, in one review, motivational/volitional strategies were favoured over environmental changes in SB intervention in reducing screen time in children and adolescents [27]. These findings have important implications for developing SB intervention strategies and components that are suitable for different age groups in different settings.

Exposure to SB is frequently operationalised under a socio-ecological system where an individual’s SB is highly influenced by multiple factors such as the nature of their tasks and the tools/ equipment used to perform the tasks [16]. It explained why sit-stand desks in the workplace setting could be very effective in reducing sitting time as sitting itself is not a prerequisite for the work at hand [44, 45].

This contrasts to other types of SB such as driving an automobile, where inherent challenges still remain in terms of addressing all the behavioural influences to promote meaningful reductions in time spent sitting. One review attempted to investigate the intervention effectiveness in reducing sitting while using public transport, but there were insufficient primary studies to conduct a meaningful evidence synthesis [39]. Future research should also focus on initiatives to explore other SB non-occupational settings such as leisure or domestic activities.

Moreover, future research efforts should be directed at unpacking the finer details of SB reduction interventions, including identifying the constituent behaviour change techniques/strategies applied and the relative effectiveness of such approaches [44–46].

## Conclusion

In conclusion, there is strong evidence supporting the effectiveness of SB interventions in reducing sedentary time, especially interventions targeting occupational sitting in office settings with a clinically meaningful reduction of at least 30 min per day [47]. SB interventions were also effective in reducing screen time in children and adolescents; however, the effect size appears to be small. Future research needs to explore the potential of SB in older age groups outside of occupational settings as well as during sedentary leisure time. Moreover, sustainability of changes in SB remains a challenge.

## Supplementary information

**Additional file 1.** Search strategy by database.

**Additional file 2.** Characteristics of included studies.

## Data Availability

All data generated or analysed during this study are included in this published article and its supplementary information files.

## Abbreviations

AMSTAR: A measure tool to assess systematic reviews
CVD: Cardiovascular disease
GRADE: Grading of recommendations, assessment, development and evaluations
JBI: Joanna Briggs Institute
MD: Mean difference
MET: Metabolic equivalents of task
PA: Physical activity
RCT: Randomised controlled trial
SB: Sedentary behaviour
SMD: Standardized mean difference
T2D: Type 2 diabetes
