# Supplementary material for: The effectiveness of sedentary behaviour interventions on sitting time and screen time in children and adults: an umbrella review of systematic reviews
Source: Int J Behav Nutr Phys Act. 2020 Sep 21;17:117. doi: 10.1186/s12966-020-01009-3 (PMC7504841; doi:10.1186/s12966-020-01009-3)
Supplement: Supplementary file 1 — Additional file 1. Search strategy by database. [file 12966_2020_1009_MOESM1_ESM.docx]

Appendix 1. Search strategy by database

Search strategies Medline Complete

| **#** | **Query** |
| --- | --- |
| S34 | S28 AND S29 AND S30 |
| S33 | S28 AND S29 AND S30 |
| S32 | S28 AND S29 AND S30 |
| S31 | S28 AND S29 AND S30 |
| S30 | S18 OR S19 OR S20 OR S21 OR S26 OR S27 |
| S29 | S12 OR S13 OR S14 OR S15 OR S16 OR S17 OR S25 |
| S28 | S1 OR S2 OR S3 OR S4 OR S5 OR S6 OR S7 OR S8 OR S9 OR S10 OR S11 OR S22 OR S23 OR S24 |
| S27 | (MH "Meta-Analysis as Topic") |
| S26 | (MH "Systematic Reviews as Topic") |
| S25 | (MH "Program Evaluation") OR (MH "Evaluation Studies") |
| S24 | (MH "Screen Time") |
| S23 | (MH "Sitting Position") |
| S22 | (MH "Sedentary Behavior") |
| S21 | TI “quantitative analys*” OR AB “quantitative analys*” |
| S20 | TI meta-analytic OR AB meta-analytic |
| S19 | TI meta-analys* OR AB meta-analys* |
| S18 | TI “systematic review” OR AB “systematic review” |
| S17 | TI compar* OR AB compar* |
| S16 | TI program* OR AB program* |
| S15 | TI intervention* OR AB intervention* |
| S14 | TI evaluat* OR AB evaluat* |
| S13 | TI efficac* OR AB efficac* |
| S12 | TI effect* OR AB effect* |
| S11 | TI inactive* OR AB inactive* |
| S10 | AB ( (watch* OR view*) N2 (TV OR television) ) OR TI ( (watch* OR view*) N2 (TV OR television) ) |
| S9 | TI “computer time” OR AB “computer time” |
| S8 | TI “screen time” OR AB “screen time” |
| S7 | TI “seated posture” OR AB “seated posture” |
| S6 | TI sitting OR AB sitting |
| S5 | TI “sedentary leisure" OR AB “sedentary leisure" |
| S4 | TI “sedentary activit*" OR AB “sedentary activit*” |
| S3 | TI “sedentary time” OR AB “sedentary time” |
| S2 | TI “sedentary lifestyle*” OR AB “sedentary lifestyle*” |
| S1 | TI "sedentary behavio*” OR AB "sedentary behavio*” |
