# Supplementary material for: The effectiveness of sedentary behaviour interventions on sitting time and screen time in children and adults: an umbrella review of systematic reviews
Source: Int J Behav Nutr Phys Act. 2020 Sep 21;17:117. doi: 10.1186/s12966-020-01009-3 (PMC7504841; doi:10.1186/s12966-020-01009-3)
Supplement: Supplementary file 2 — Additional file 2. Characteristics of included studies. [file 12966_2020_1009_MOESM2_ESM.docx]

Appendix 2: Characteristics of included studies

Reviews of sedentary behaviour interventions in children and adolescents

| **Author/**  **Year**  **(Quality assess-ment)*** | **Objectives of the review** | **Number of studies included in the review;**  **Sample size;**  **Age group** | **Databases and search and quality assessment (QA) tool** | **Description of primary interventions** | **Sedentary behaviour outcomes &**  **Outcome measurement** | **Meta-analysis results** |
| --- | --- | --- | --- | --- | --- | --- |
| Biddle  2011  (Low) | To assess the effectiveness of SB intervention in young people | **Number of studies included**: N = 17 (controlled trials)  **Total sample size** N = 4976, ranged from 10 to 1295  **Age**: 18 years and younger | **Databases**: N = 5 (ERIC, MedLine, PsychInfo, SportDiscus and the Cochrane Library databases)  **Date of search**: 2010  **Publication date range**: 1999 - 2008  **QA**: was not conducted  **Meta-analysis** was conducted with a random effect model using Hedges’ g to report a standardized effect size estimates. | **Settings**: Community (n=4), Education setting (n=9), Clinic (=3), laboratory (n= 1)  **Country**: USA (n = 11), UK (N=2), France (n = 1), Canada (n=1) and Australia (n = 2).  **Length of intervention**: ranged from 1 to 4 months  **Intervention components:** interventions components of primary studies were not reported in the review. The majority (n=16) were multi-risk behaviour interventions that targeted sedentary, PA and/or nutrition.  **Controls:** not reported in this review | **SB outcome**: TV viewing or screen time/ day **Other outcomes**: N/A  **Measurement of sedentary time** : self-report (n=7), objective measure (n=3), combined self-report and objective measure (n=7) | - Primary analysis of all included studies revealed a small but significant change in SB favouring intervention: (g = − 0.192; SE = 0.056; 95% CI = −0.303 to −0.082; p = 0.001) with a significant heterogeneous distribution (QT=43.48, p < 0.001; I^2^ = 63.21). - Subgroups analysis reported a significant reduction of SB following the community-based interventions, and interventions that deliver less than 4 months or longer than 12 months. |
| Downing 2018  (Moderate) | To examine the effectiveness of behavioural interventions to reduce SB in children | **Number of studies included:** 31 studies (2 RCTs, 29 cluster RCTs)  **Total sample size:** N=10,655, ranged 22 to 885  **Age**: 3 to 5 years | **Databases**: N = 7 (EBSCOhost  (Academic Search Complete, CINAHL Complete, Global  Health, MEDLINE Complete, PsycINFO, SPORTDiscus with  Full Text) and EMBASE)  **Date of search**: March 2016  **Publication date range**: 2004 to 2016  **QA**: EPHPP tool for quantitative studies  **Meta-analysis** reported between groups mean difference (MD), using the generic inverse variance method with random-effect model.  No publication bias assessment was conducted. | **Settings**: preschool/ kindergarten/ day-care centres (n=18), home-based (n=7), primary care (n=3) and community (n=3)  **Country:** USA (n=15), Australia (n= 5), Belgium (n= 3), UK (n=2), one each in Canada, Netherland, Switzerland, German, Israel, Turkey. **Length of intervention**: 1 off session - 24 months  **Intervention components^*^:**   - Preschool/day-care setting: educational sessions, changes in school policy (screen time, PA), newsletters to parents. - Home setting: trained nurse provided counselling, educational sessions or material to parents. - Primary care: One-off session on a healthy lifestyle; follow-up phone calls. - Community: interventions delivered to parents with guidance around diet, physical activity and screen time.   Note: all seven studies that reported continuous measures of sedentary time took place in preschool settings.  **Comparator**: not reported in the review | **SB outcome**: Sedentary time (n=8) and screen time (n=18) as a proxy measure of SB  **Other outcomes**: N/A  **Measurement of sedentary time**: objectively measures (n=12) and observation report by parents or educators. | - Meta-analysis of sedentary time (n=7 studies) reported a pooled MD of -18.91 minutes per day (95% CI −33.31 to −4.51); I^2=^ 89% - Meta-analysis of screen time (n=17) reported the between groups MD of −17.12 (95% CI −28.82 to −5.42); - When sedentary time and screen time were pooled together, the analysis showed a reduction of −17.76 (95% CI −26.90 to −8.62) minutes per day. |
| Kamath 2008  (Low) | To assess the effectiveness of intervention preventing childhood obesity in changing lifestyle including SB | **Number of studies included:** 36 RCTs  **Total sample size:** N= 9891, ranged not reported  **Age:** 2 to 18 years | **Databases:** N= 5 (MEDLINE, ERIC, EMBASE, CINHAL, PSYCInfo, DISSERTATION  abstracts, Science Citation Index, Social Science Citation Index,  and the Cochrane Database of controlled clinical trials)  **Date of search**: February 2006  **Publication date range**: 1988 to 2004  **QA**: a non-validated tool  **Meta-analysis:** (n= 36) reports between groups standardised mean different. | **Settings**: home, school, clinic, or community setting.  **Country**: not reported  **Length of intervention**: 4 weeks to 3 years  **Intervention components related to SB:**   - Cognitive and Behavioural training target SB: goal setting in screen time reduction; monitoring and reinforcement for behaviour change - Environmental change at hone to limit television watching and screen time - Teacher and parental support using activities guide.   **Comparator**: not reported in the review. | **SB outcome**: Sedentary activity as screen time (n=14)  **Other outcomes**: N/A  **Measurement of sedentary time**: both self-report/ proxy report (by teacher and parent) and objective measure. | - Meta-analysis of sedentary activities measure as hours of screen time per day (n=14 studies) reported a pooled SMD of -0.29 (95% CI -0.25 to -0.22); I^2=^ 0%. - Sub-group analysis indicated that the effect size is larger in young children than adolescent; - Trials that conducted more than 6 month were likely to be more effective than short trials (less than 6 months) |
| Grieken 2012  (Low) | To synthesis the evidence regarding the effects of interventions preventing SB in children and adolescents. | **Number of studies included**: N = 34 (30 RCTs, 3 controlled trials)  **Total sample size**: not reported  **Age**: 0 to 18 years | **Databases**: N = 5 (PubMed, EMBASE, Web of Science, PsycINFO and Cochrane Database of Systematic Review)  **Date of search**: March 2011  **Publication date range**: 1990 - 2011  **QA**: Cochrane RoB tool  **Meta-analysis** reported both pre- and post and between groups mean difference in SB and BMI using random effect model. | **Settings**: School based (n= 14), primary care (n= 2), home based (n= 4), community (n=1), multi-settings (n=13)  **Country**: USA (n = 20), UK (n = 3), Europe (n = 5), Asia (n = 3) and Australia (n = 3), Mexico (n=1), New Zealand (n=2)  **Length of intervention**: 5 days to 4 years  **Intervention components**:   - Educational strategies: educational session or material for parents and/ or teachers regarding screen time reduction. - Behaviour changes: goal setting, screen time budget hours, rules and boundaries regarding TV viewing - Environmental changes: TV monitor or moving TV to another room. - Promote other type of activities as a replacement of screen time/ screen leisure in both home and school setting. - Curriculum changes at school   **Comparator**: no intervention (n =26), educational sessions (n=6), counselling (n=1), PA (n=2) | **SB outcome**: sedentary screen time in minutes per day (n=33) **Other outcome**: Anthropometric  **Measurement of sedentary time**: Parent self-report/ child self-report (n=30); objective measure (n=3). | - Meta-analysis of all included studies using random-effect model reported a post-intervention mean reduction of 17.95 (95% CI 9.28 to 26.61) minutes per day; - Post-intervention change from baseline were -20.44 (95% CI −30.69;–10.20) - Sub-groups analysis reveal no significant difference between single and multiple components interventions. |
| Maniccia 2011  (Low) | To investigate the effectiveness of intervention targeted children’s screen time | **Number of studies included**: N = 33 (RCTs=23, and others n=6)  **Total sample size** not reported; ranged from 2 to 954  **Age**: ranged 5 to 18  8 studies targeted obese children. | **Databases**: N = 14, including Cochrane library, Medline, PsychInfo  **Date of search**: 24 Nov-6 Dec 2008  **Publication date range**: 1997 - 2008  **QA**: not conducted  **Meta-analysis** (n = 29) reported pooled standardized mean difference (SMD) and Hedges g using random effect method. Publication bias assessment was conducted. | **Settings**: school (n=13), home (n=7) and other settings (n=8)  **Country^*^:** USA (n=20), EU (n=4), UK (n=1), Canada (n=1) Israel (n=1) and Australia (n=1). **Length of intervention**: not reported  **Intervention components^*^:** only 4 studies targeted sole SB, 5 studies included both SB and PA and the other were multifaceted intervention (included diet, PA and SB).  Common SB components included:   - Environmental changes (n=9): limiting physical access to screen devices, TV control devices - Behaviour changes: awareness session at school; Budgeting screen time sessions, groups-based educational workshop, and activity monitoring; bookmark, vouchers etc.   **Comparator**: control (n=16), other intervention (n=2) | **SB outcome:** the primary outcome was screen time (n=19)  **Other SB outcome**: eating while watching/ having TV in bed room (n=5).  **Measurement of sedentary time:** not reported | - The primary analysis reported overall SMD of -0.148 (95%CI -0.224 to -0.071) and Hedges g of -0.144 (95% CI: -0.217 to -0.072) - Subgroups analysis revealed a non-significant effect size followed home-based interventions and overweight population. - Pooled effect size of 5-11 years (n=18) was slightly smaller than 12-18 years (n=6): -0.125 (95%CI -0.241 to -0.008) vs -0.176 (95% CI -0.304 to -0.049) - Subgroups analysis comparing intervention vs do-nothing control reported a large significant effect size favouring intervention after adjustment: -0.243 (95%CI -0.401 to -0.085); and non-significant results when different interventions were compared. |
| Wahi  2018  (Moderate) | evaluate the impact of interventions  aimed at reducing screen time | **Number of studies included**: N = 13 (controlled trials)  **Total sample size** N = 3133, ranged from 21 to 1295  **Age**: ranged from 3.9 to 11.7 | **Databases**: N = 6 (Medline, EMBASE, CENTRAL, Psycinfo, ERIC and CINAHL)  **Date of search**: April 21, 2011.  **Publication date range**: 1948-2017  **QA**: Cochrane RoB and GRADE  **Meta-analysis** reported between groups unadjusted difference in mean (SEM) change in screen time using the generic inverse method, random-effects model. | **Settings**: school, community and specialty clinic  **Country^*^:** USA (n=11), UK (n=1), New Zealand (n=1) **Length of intervention**: 1- 24 months  **Intervention components:**   - Class-room based curriculum (health promotion or Planet Health (n=4) - Individual counselling for children and parent/family (n=3) - Automated monitor controlling screen time (n=4) - Intervention mapping workshop and newsletter/seminar (n=2). - Home-based screen time reduction intervention (n=1)   **Comparator:** not reported in the review. | **SB outcome:** Sedentary time including screen time, television view per hour per week (n=9).  **Measurement of sedentary time:** self-reported screen time. | - Meta-analysis indicated a non-statistically significant reduction in screen time MD=-0.90 h/wk (95% CI −3.47 to 1.66 h/wk) (*P*=0.49), I^2^=66%, *P*=.003 - A subgroup analysis of preschool children reported MD of −3.72 h/wk (95% CI, −7.23 to −0.20 h/wk) (P=.04). |
| Note: * The number of intervention strategies/ components are not necessary to add-up due to the number of multi-components studies and multiple arm comparisons.  BMI: body max index; CI: confident interval; EPHPP: Effective Public Health Practice Project; GRADE: Grading of Recommendations Assessments, Developments and Evaluation; MD: mean difference; N/A: not available; PA: physical activity; QA: Quality assessment; SB: sedentary behaviour; RCT: randomized controlled trial; RoB: Cochrane Risk of Bias tool. | | | | | | |

Reviews of sedentary behaviour interventions in adults

| **Author/**  **Year**  **(Quality assess-ment)*** | **Objectives of the review included** | **Number of studies included in the review;**  **Sample size** | **Databases and search and quality assessment (QA) tool** | **Description of primary interventions** | **Sedentary behaviour outcomes &**  **Outcome measurement** | **Meta-analysis results** |
| --- | --- | --- | --- | --- | --- | --- |
| Comper-nolle  2019  (Moderate) | To assess the effectiveness of interventions using self-monitoring to reduce SB in adults. | **Number of studies included**: N = 19 (RCTs n=11)  **Total sample size** N = 2800, ranged from 23 to 1113  **Age**: 18 and above  5 studies involved overweight and obese people; T2D, breast cancer and MS one study each. | **Databases**: N = 5 (PubMed, Embase, Web of Science, and The Cochrane Library)  **Date of search**: October 2018 and updated May 2019  **Publication date range**: 2012 - 2019  **QA**: EPHPP tool  **Meta-analysis:** N = 18 studies; effect size was calculated with Hedges’g, using random-effect model.  Publication bias assessment was conducted. | **Settings**: not reported  **Country^*^:** USA (n=6), the UK (n=4), Belgium (n=3), Australia (n=2), Canada (n=1), Japan (n=1) and Taiwan (n=1).  **Length of intervention**: from 1 week to 1 year  **Intervention components^*^:** 12 intervention targeted sole SB, 6 studies combined SB + PA and one was multifaceted intervention with SB + PA + diet.  Self-monitoring component included:   - Use of monitoring devices/ logbook/ questionnaire about office sitting, leisure sitting - Combined with behaviour changes techniques   **Comparator:** do-nothing control (n=14); behaviour techniques without monitoring devices (n=3) | **SB outcome:** general sedentary time  **Other SB outcome**:  **Measurement of sedentary time:** self-report (n=3) and objective measure (n=13: Jawbone Up 24, accelerometer, pedometer, Fitbit etc.) and combined (n=3) | - Self-monitoring significantly reduced total sedentary time (Hedges g = 0,32; 95%CI 0,14 to 0,50; p = 0,001) and occupational sedentary time (Hedge’s g = 0,56; 95% CI 0,07 to 0,90; p =0,02) - Intervention that focus sole on SB had larger effect size in comparison to intervention with PA and or diet: g =0.45 (95%CI 0,15 to 0,75) vs g=0.16 (95% CI 0,001 to 0,31) - Sub-group analysis revealed a non-significant effect size following subjective monitoring (log book, diaries) while objective monitoring devices offered a large significant effect size g= 0,40 (95%CI 0,19 to 0,60) |
| Chu 2016  (Moderate) | To investigate the effectiveness of workplace interventions for reducing prolonged sitting among office workers | **Number of studies included: N=**26 (15 RCTs and 11 non-RCTs)  **Total sample size** N = 4568, ranged 20 to 787  **Age**: 33.5 to 50.4 years | **Databases**: N = 5 (PubMed, Cochrane  Central Register of Controlled Trials, Scopus, Web of Science  and Canadian Centre for Occupational Safety and  Health reference)  **Date of search**: December 2015  **Publication date range**: 2003 to 2015  **QA**: Cochrane RoB tool  **Meta-analysis** reported between groups mean difference, using the inverse variance method with both fix and random effect model. Publication bias assessment was conducted. | **Settings**: office setting (n = 26).  **Country^*^**: Australia (n = 8), UK (n = 3), USA (n = 6), Europe (n = 10).  **Length of intervention**: 5 days - 14 months  **Intervention components:**   - Educational/behavioural change: motivational interview, goal setting, action planning, prompt behaviour changes. - Environmental change: Sit-stand workstation, portable elliptical/pedal machine; stationary cycle ergometer and treadmill desk. - Multi-component: environmental changes in combination with behavioural interventions.   **Comparator:** no intervention controls | **SB outcome**: sitting time (minute/ 8-hour workday or minute/ waking hours); sitting energy expenditure (MET-minutes or calories)  **Other outcomes**: N/A  **Measurement of sedentary time:** self-report (n = 14); objective measurement (n = 12) | - The pooled effect size (21 studies) showed a significant reduction in workplace sitting of 39.6 min/8-hour workdays (95% CI: 51.7, 27.5), favouring the intervention group. - Multi-component interventions (5 studies) reported the largest effect size of -88.8 min/8-hour workday; 95% CI: -132.7, -44.9), followed by environmental (6 studies) with a reduction of 72.8 min/8-hour workday; 95% CI: 104.9, 40.6) and then Educational/ behavioural strategies (15 studies) had a reduction of 15.5 min/8-hour workday (95% CI: 22.9, 8.2). - Sub-group analysis compared study designs showed similar results between RCTs and non-RCTs. - Sub-group analysis compared objective and self-reported measures indicated a greater effect size following objective measures (-66.7 min/8 hours versus -23.3 min/8 hours in self-report). - Sub-group analysis revealed that good- quality studies had slightly higher effect size (-48.3 min/ 8 hours) than poor-quality studies (-36.6 min/ 8 hours). |
| Neuhaus 2014  (Moderate) | To investigate the effectiveness of activity-permissive workstation in reducing occupational sedentary time | **Number of studies included**: N = 38 (RCTs=9, pre-post n=13, crossover n=16)  **Total sample size** N = 984, ranged from 2 to 66  **Age**: not reported in the review | **Databases**: N = 9 (Web of Knowledge, Medline, Embase, CINAHL, SPORTDiscus, CENTRAL, Scopus, PsychInfo  and AMED)  **Date of search**: July 2013  **Publication date range**: 1996 - 2013  **QA**: Cochrane RoB tool  **Meta-analysis** reported between groups standardized difference in workplace sedentary time, using random effect method. No publication bias assessment was conducted. | **Settings**: Laboratory experiment (n = 18) and office setting (n = 20).  **Country**: North America (n = 23), Europe (n = 4), Asia (n = 3) and Australia (n = 8).  **Length of intervention**: 1 day - 12 months (Office-based intervention had mean duration of 15 weeks)  **Intervention components^*^**:   - Height-adjustable desks (n = 17), standing desks with height-adjustable chairs (n = 2), standing desks without height-adjustable chairs (n = 8), treadmill desks (n = 12), pedal devices (n = 2), cycle ergometers (n = 2), a stepping device (n = 1), both treadmills and cycle ergometers (n = 1). - Office-based interventions had additional strategies, i.e. motivational messages, instruction to stand/ break prolonged sitting.   **Comparator**: not reported in the review | **SB outcome**: sitting time/8-hour workday (reported in 13 studies – 14 comparisons) **Other outcomes**: other health-related outcomes (n = 23): BMI, weight, blood profile; Work-related outcomes (n=23): performance, productivity, absenteeism and presentism.  **Measurement of sedentary time (n=14 comparisons)**: 11 studies used an objective method to measure sedentary time; 3 studies used self-report | - Meta-analysis of 8 comparisons (from 7 office-based studies) reported a reduction in sitting time of −77 min/ 8-hour workday (95% CI −120 to −35); I^2^ = 91%.   No sub-group analysis was conducted. |
| Martin 2015  (Low) | To assess the intervention effectiveness in reducing sedentary time in adults | **Number of studies included**: N = 51 (RCTs n = 44, cluster RCTs n=7)  **Total sample size** N=18,480, ranged not reported.  **Age**: 18 and above  Condition: overweight or obese (n=5), T2D (n=5), CVDs (n=3). | **Databases**: N = 13 (including CENTRAL, Medline, Embase, PsycINFO, SPORTDiscus, CINAHL etc.)  **Date of search**: January 2014  **Publication date range**:  **QA**: Cochrane RoB tool and GRADE  **Meta-analysis** (n=34): Effect sizes were calculated as between groups mean differences (min/day) using random-effect model.  Publication bias was conducted | **Settings**: work place (n=8), home/community  **Country^*^:** Europe (n=25), the USA (n=18),  Australia (n=7) and China (n=1).  **Length of intervention**: 4 days to 3 years  **Intervention components^*^:** SB interventions (n=3), PA intervention (n=9), SB+PA intervention (n=16) and multi-risk behaviour studies SB + PA + Diet (n=20). Common SB components included:   - Educational strategies/ counselling: face-to-face or phone counselling session, educational session, self-monitoring strategies, goal setting,   **Comparator:** no intervention (n=7), attention control (n=5), waiting list (n=7), usual care (n= 10), alternative intervention (n=20) | **SB outcome**: time spend in SB in minute per day (n=49), number of sitting breaks (n=3), number of prolonged sitting event (n=3)  **Other SB outcome**: none  **Measurement of sedentary time:** self-report (n=31), objective measure (n=6) | - Overall reduction in sedentary time of MD: −22.34 min/day (95% CI −35.81 to −8.88, I2=71%) favouring the intervention. - SB interventions (n=2, 62 participants) yielded an MD of −41.76 min/ day (95% CI −78.92 to −4.60, p=0.003, I2=65%). While the reduction in sedentary time of PA and PA+SB interventions were non-significant. - Interventions that delivered between 3-6 months tended to be more effective than short term and long term interventions. |
| Peachey 2018  (Moderate) | To investigate the effectiveness of intervention reducing SB in adults and compare different intervention components. | **Number of studies included**: N = 38 (RCTs=24, cluster RTs n=7, quasi-RT n =1 and others n=6)  **Total sample size** N = 5983, ranged from 10 to 1480  **Age**: ranged 18 to 70  9 studies involved overweight and obese people. | **Databases**: N = 5 (Medline, CINAHL, SPORTDiscus, PsychInfo and PubMed)  **Date of search**: 2017  **Publication date range**: 2003 - 2017  **QA**: Cochrane RoB tool  **Meta-analysis** (n = 35) reported pooled intervention effects (MD in min per day) using random effect method. Publication bias assessment was conducted. | **Settings**: office setting (n=17), community (n=5), multi-settings (n=12) and home (n=1)  **Country^*^:** USA (n=13), EU (n=12), UK (n=3), Canada (n=1) and Australia (n=9). **Length of intervention**: 1 week to 2 years  **Intervention components^*^:**   - Environmental changes (n = 12): replace the conventional desk with other type of work desks (standing desk, sit-stand desk, vertical workstations on treadmills, desk cycle/cycling desks, or stepping devices) and/or computer prompts; TV locking device. - Behaviour changes (n=20): counselling sessions, groups-based educational workshop, and activity monitoring. - Multi-component interventions (n = 8): included both environmental and behaviour changes components.   **Comparator**: No intervention (n= 13), wait-list control (n=3) and other interventions as control (n=22). | **SB outcome:** overall daily sitting time in  minutes or hours per day (n=18) or per week (n=6), sitting minutes per 8 hour workday (n=6), percentage of the assessed time period (n=5), television viewing hours per day (n=2)  **Other SB outcome**: energy expenditure from sitting (n=1).  **Measurement of sedentary time:** Objective measure (n=19) and self-report (n=19) | - The primary analysis reported a significant reduction in daily sitting time of −30.37 min/day (95% CI −40.86 to −19.89) favouring the intervention group. - Reductions in sitting time were similar between workplace (−29.96 min/day; 95% CI −44.05 to –15.87) and other settings (−30.47 min/day; 95% CI −44.68 to –16.26). - Environmental interventions had the most substantial reduction in daily sitting time (−40.59 min/day; 95% CI −61.65 to –19.53), followed by multicomponent (−35.53 min/day; 95% CI −57.27 to –13.79) and behavioural (−23.87 min/day; 95% CI −37.24 to –10.49) interventions. - Larger effect size followed self-report (-36.01 min) in comparison to objective measure (-24.63 min) |
| Prince 2014  (High) | To compare PA and SB intervention in reducing sedentary time in adults | **Number of studies included**: N = 63 (RCTs n=54, non-RCT n =9)  **Total sample size** N = 25,446; ranged from 17 to 12,287  **Age**: 18 to 94  Condition: overweight and obese (n=2); T2D (n=3), CVDs (n=2). | **Databases**: N = 7 (Medline, Embase, EBM Reviews, CENTRAL, PsycINFO, SportDiscuss, and Dissertations  and Theses)  **Date of search**: October –November 2013  **Publication date range**: 2004 to 2014  **QA**: Cochrane RoB tool and GRADE  **Meta-analysis** (n=33) reported between groups SMD (min/day) using random-effect model.  Publication bias assessment was conducted. | **Settings of SB interventions**: office, home-based, workplace and home combined (number not reported)  **Country^*^:** USA (n=21), the UK (n=5), Australia (n=12), Canada (n=2), South Korea (n=1), EU (n=4), China (n=1)  **Length of intervention**: 4 weeks to 3 years  **Intervention components^*^:** 43 PA studies, 14  PA + SB studies and 8 SB-only studies. SB components included:   - Environmental changes: sit-stand workstation, pedal machine, shared tread mill; TV controls - Other organizational and individual behaviour reinforcement components: health coach, counselling, monitoring etc.   **Comparator:** control group or usual care | **SB outcome:** sitting time (min/8-h workday), screen time (min/day), TV viewing time (mind/d or hr/week)  **Other SB outcome**: none  **Measurement of sedentary time:** self-report (n=20), objective measure (n=37) | - Meta-analysis of SB interventions yielded a significant and large reduction in sedentary time SMD = −1.28 (95% CI: −1.68 to −0.87) equating to a mean difference of 91 min/d. - SB + PA interventions resulted in a significant,   but small reduction in sedentary time: SMD = −0.37 (95% CI: −0.69, −0.05) equating to a mean difference of approximately 35 min/d   - PA interventions resulted in a significant, but small reduction in sedentary time (SMD = −0.22 (95% CI: −0.35, −0.10); equal to about -19 min/d. |
| Shrestha 2018  (High) | To examine the effectiveness of workplace interventions to reduce sitting time | **Number of studies included:** 34 studies (17 RCTs, 7 cluster- RCTs, 8 controlled before-and-after studies)  **Total sample size:** N = 3397, ranged 16 to 523  **Age**: 20 to 60 years | **Databases**: N = 8 (Cochrane  Central Register of Controlled Trials, Medline, CINAHL, Occupational Safety and Health database, PsycINFO, Embase, ClinicalTrials.gov, World Health Organization (WHO) International Clinical  Trials Registry Platform (ICTRP) search portal)  **Date of search**: August 2017  **Publication date range**:  **QA**: Cochrane RoB tool  **Meta-analysis** reported between groups mean difference (MD), using the generic inverse variance method with random-effect model.  Publication bias assessment was conducted. | **Settings**: office setting, occupational physio clinic (n=1)  **Country^*^:** Australia (n= 12), USA (n=9), Canada (n=1), UK (n=3), Netherland (n=3), Spain (n=2), Denmark (n = 1), Belgium (n= 1), Switzerland (n=1), German (n=1), Finland (n=1). **Length of intervention**: 10 days - 12 months  **Intervention components^*^:**   - Environmental changes in workplaces (n = 16): changing layout of the workplace to promote walking; replace conventional desks with new type of work desks (standing desk, sit-stand desk, vertical workstations on treadmills, desk cycle/cycling desks, or stepping devices) - Workplace policy changes (n = 4): Walking or standing meeting, breaks and stand up, sitting diaries. - Information and counselling (n = 11): counselling, motivational posters/ mails/ messages, educational leaflets. - Multi-component interventions (n = 4): include strategies from all three components above.   **Comparator**: No intervention (n= 17), sit-desk (compared with permissive workstation n=8), educational session/ information (n=4), different breaks pattern (n=2), different type of prompts/ messages (n=2), usual care (n=1). | **SB outcome**: Sitting time at work (minutes per 8-hour workday), total sitting time, prolonged sitting bouts; Standing time and stepping at work. **Other outcomes**: Energy expenditure, work productivity and adverse events.  **Measurement: of sedentary time**: Mainly objective measurement. Self-report sedentary time were used in 5 studies | - Physical workplace changes (10 studies) reduced sitting time by 100 minutes/workday at short-term follow-up (up to three months) compared to sit-desks (95% CI −116 to −84), but the quality of evidence was low. - Primary studies reported a non-significant reduction following workplace policy changes: MD −15 minutes/ day (95% CI −50 to 19) at short term; and MD −17 minutes/day (95% CI −61 to 28) at medium-term follow-up. - Information and counselling interventions (2 studies) reported a non-significant change in time spent sitting at work at short-term follow-up (MD −19 minutes per day, 95% CI −57 to 19. However, the reduction was significant at medium-term follow-up (MD −28 minutes per day, 95% CI −51 to −5). - Meta-analysis of three multi-components studies showed a MD of -45.60 minutes per day, 95% CI -62.54 to -28.66 at medium-term follow-up. |
| Shrestha 2019  (High) | To assess the effectiveness of SB intervention in non-occupational settings | **Number of studies included**: N = 19 (RCTs=12, cross-over RCTs n=2, cluster RCT n =5)  **Total sample size** not reported in the review  **Age**: ranged from 18 to 69  Involve people with conditions: T2D or at risk of T2D (n=3), obese (n=2), at risk of CVD (n= 1) | **Databases**: N = 9 (Academic Search Premier, Nursing/ Academic Edition of Health Source, MasterFILE  Premier, SPORTDiscus, MEDLINE/PubMed, Scopus, PsycINFO, CINAHL and Web of Science)  **Date of search**: 19 October 2016  **Publication date range**: 2009 - 2017  **QA**: Cochrane RoB tool  **Meta-analysis** (n = 14) calculated the pooled effect size of mean differences between intervention and control using random-effect model  Publication bias assessment was not conducted. | **Settings**: office setting (n=4), community (n=5), home (n=5), and others (n=10)  **Country^*^:** USA (n=10), EU (n=6), Australia (n=2), China (n=1) **Length of intervention**: 2 weeks to 2 years  **Intervention components^*^:**   - Environmental changes (n = 6): replace conventional desks with new type of work desks (standing desk, sit-stand desk, vertical workstations on treadmills, desk cycle/cycling desks, or stepping devices) and/or computer prompts; TV locking device. - Behaviour changes (n=16): counselling sessions, groups-based educational workshop, and activity monitoring. - Multi-component interventions (n = 19): included both environmental and behaviour changes components.   **Comparator:** usual lifestyle/ usual care (n=10), self-help with minimum support (n=6) and other interventions (n=3). | **SB outcomes:** TV viewing (n=10), leisure sitting times (n=9), leisure computer use (n=4), transport sitting (n= 3).  **Other SB outcomes:** none  **Measurement of sedentary time^*^**: self-report (questionnaires n=13), TV control devices (n=2), accelerometers and self-report combined (n=3) | - Meta-analysis of total leisure sitting time (n=6) reported a reduction of 30 min/day (95% CI −58 to −2 min/day), I^2^ = 91%. - The pooled effect size for TV viewing was 61 min per day (95% CI −79 to –43; I2=79%) favouring interventions at short term follow-up and 11 min per day (95% CI −20 to –2; I2=49%) at medium-term follow-up. - Analyses of leisure computer use and transport sitting both reported non-significant reductions. |
| Stephenson 2017  (Moderate) | To examine the effectiveness of technology interventions to reduce SB in healthy adults | **Number of studies included:** N=17 (1 Cluster RCT, 2 cross-over RCTs, 14 RCTs)  **Total sample size:** N=1967, ranged  **Age**: 18 to 40 years | **Databases**: N = 5 (Medline, Embase, CINAHL, PsycINFO and PubMed)  **Date of search**: June 2016  **Publication date range**: 2012 to 2016  **QA**: Cochrane RoB tool  **Meta-analysis** reported between groups mean difference (MD), using both fix and random effect model.  No publication bias assessment was conducted. | **Settings:**  workplace (n=10), community and home-based (n=5), workplace and community/ home-based (n=2)  **Country:** N/A  **Length of intervention**: 5 days to 24 months  **Intervention components:**  11 studies targeted SB alone, 3 studies had both SB and PA and the other three studies had multiple risk behaviours.   - All intervention had technology components: computer, mobile, wearable devices, software or computer prompts; emails; websites to relay information and provide feedback to participants; and text messages. - Other common components are group educational sessions, counselling. - Environmental changes: sit-stand desk, pedal machine.   **Comparator:**  no intervention (n= 8); waitlist (n=1); health information (n=7); standing desk (compared with standing desk plus computer prompts; n=1) | **SB outcome**: Sedentary time (n=14) as minutes per day and screen time as a proxy measure of SB (n=3)  **Other outcomes**: N/A  **Measurement: of sedentary time^*^**: objectively measures (n=11) and self-reported (n=9). | - Meta-analysis of 15 studies reported an overall reduction of sitting time of −41.28 min/day (95% CI -60.99 − 21.58, I^2^ = 77%, n = 1402). - Subgroup analysis of objective measures of SB reported a MD of −35.07 min/day (95%CI -46.57, −23.57, I^2^ = 21%, n = 595), favouring of the intervention group. - Subjective measures reported MD of -52.66 min/day (95% CI -93.63 to -11.69), I^2^ 88% - Interventions targeting overall daily sitting (7 studies) reported a pooled MD of −45.11 min/day (95% CI -86.63, −3.60, I^2^ = 82%, n = 640), favouring the intervention group. |
| Note: * The number of intervention strategies/ components are not necessary mutual add-up due to the number of multi-components studies and multiple arms comparisons.  BMI: body max index; CI: confident interval; CVD: cardiovascular diseases; EPHPP: Effective Public Health Practice Project; GRADE: Grading of Recommendations Assessments, Developments and Evaluation; MD: mean difference; N/A: not available; PA: physical activity; QA: Quality assessment; SB: sedentary behaviour; RCT: randomized controlled trial; T2D: type 2 diabetes; RoB: Cochrane Risk of Bias tool. | | | | | | |

Reviews of sedentary behaviour interventions with mix-age groups

| **Author/**  **Year**  **(Quality assess-ment)*** | **Objectives of the review included** | **Number of studies included in the review;**  **Sample size** | **Databases and search and quality assessment (QA) tool** | **Description of primary interventions** | **Sedentary behaviour outcomes &**  **Outcome measurement** | **Meta-analysis results** |
| --- | --- | --- | --- | --- | --- | --- |
| Direito et al 2017  (Low) | To compare the effectiveness of mobile technologies interventions to promote PA and reduce SB with usual care | **Number of studies included**: N = 21 (controlled trials)  **Total sample size** N = 1701, ranged 20 to 301  **Age**: median age 40.1, ranged from 8.4 to 71.7 | **Databases**: N = 7 (CENTRAL, CINAHL, Embase, MEDLINE, PsycINFO, Web of Science and PubMed)  **Date of search**: Jan 2015  **Publication date range**: 2007 - 2014  **QA**: Cochrane RoB tool  **Meta-analysis** reported between groups standardized mean difference (SMD) in sedentary behaviour duration | **Settings**: various.  **Country**: USA (n = 11), UK (N=3), Europe (n = 3), Canada (n=1) and Australia (n = 3).  **Length of intervention**: 1 week – 52 weeks, mean duration of 9 weeks  **Intervention components**: using mobile technologies such as mobile phone, personal digital assistant and other wireless devices; application and messaging services to promote PA and reduce SB with or without cognitive behavioural component.  **Controls** included non-technology materials such as print out/ counselling or do-nothing. | **SB outcome**: sitting time/day, screen time/ day **Other outcomes**: Bodyweight  **Measurement of sedentary time** (n=5 comparisons): 1 studies used an objective method to measure sedentary time; 3 studies used self-report | Meta-analysis of 5 include studies reported a reduction in SB: SMD -0.26 (95% CI -0.53 to -0.00), I^2^ = 0%.  Subgroup analysis reported non-significant results for both objective measures (SMD−0.24, 95 % CI−1.00 to 0.52) and self-report (SMD −0.27, 95 % CI−0.55 to 0.01). |
| Wu et al 2016  Moderate | To assess the effectiveness of intervention targeting screen time | **Number of studies included:** N=14 RCTs  **Total sample size:** N=2238, ranged from 21 to 475  **Age**: 3 to 54 years | **Databases**: N = 3 (The PubMed, Embase, and CENTRAL)  **Date of search**: 24 August 2015  **Publication date range**: 1999 to 2015  **QA**: Cochrane Collaboration’s Risk of Bias tool  **Meta-analysis** reported between groups mean difference (MD), using random-effect model.  No publication bias assessment was conducted. | **Settings:**  Kindergarten/schools (n=3), community (n = 7) and clinics (n=4)  **Country:** Canada (n = 1), U.S.(N = 10), New Zealand (n=2), Turkey (n=1)  **Length of intervention**: 3 weeks to 24 months  **Intervention components:**   - Automated monitor TV viewing - Telephone counselling - Classroom-based health promotion and change of curriculum.   **Comparator:**  No intervention (n=11); Physical activity (n=1); verbal advice (versus automated TV monitor, n=1); counselling (n=1). | **SB outcome**: TV viewing (n=3) was measured in hours per week (excluded computer time, video games); screen time in total (n=11).  **Other outcomes**: n/a  **Measurement: of sedentary time^*^**: objectively measures (n=1) and self-reported (n=13). | - Meta-analysis if all included studies reported a reduction in screen time MD -4.63 hrs/week (95% CI -7.68 to -1.59), I^2^ = 94.6%. - Subgroup analysis indicated that health promotion and counselling intervention (MD -8.76 (95% CI -14.33; -3.19) were likely to be more effective than automated monitor (MD -1.99, 95% CI -5.56; 1.59).. - Interventions that were less than 7 months had larger effect size in reduction of screen time than longer intervention: -8.94 (95% CI -13.17; -4.71) and -1.43 (95% CI -3.50; 0.64) respectively. |
| Note: * The number of intervention strategies/ components are not necessary mutual add-up due to the number of multi-components studies and multiple arms comparisons.  BMI: body max index; BS: sedentary behaviour; CI: confident interval; EPHPP: Effective Public Health Practice Project; MD: mean difference; N/A: not available; PA: physical activity; QA: Quality assessment; SB: sedentary behaviour; RCT: randomized controlled trial; RoB: Cochrane Risk of Bias tool. | | | | | | |
